# Identification of *EPHB4* as a potential causal gene and therapeutic target for endometriosis using Mendelian randomization

**DOI:** 10.1186/s41065-025-00457-w

**Published:** 2025-05-31

**Authors:** Shaohua Ling, Delong Xie, Lifang Huang, Siqi Huang, Chun Tian, Liying Huang, Rong Chen, Li Qin, Xiao Qin

**Affiliations:** 1 Reproductive Medicine Center, Baise People’s Hospital/Affiliated Southwest Hospital of Youjiang Medical University for Nationalities, Baise, China; 2 Reproductive Medicine Center, Affiliated Hospital of Youjiang Medical University for Nationalities, Baise, China; 3 Clinical Laboratory, Baise People’s Hospital/Affiliated Southwest Hospital of Youjiang Medical University for Nationalities, Baise, China; 4 Department of Gynecology, Baise People’s Hospital/Affiliated Southwest Hospital of Youjiang Medical University for Nationalities, Baise, China

**Keywords:** Endometriosis, Mendelian randomization, Druggable genome, *EPHB4*, Colocalization analysis

## Abstract

**Objectives:**

Endometriosis is a common condition among women, characterized by chronic pain and infertility, presenting significant challenges for clinicians. This study aims to identify potential druggable targets to offer new therapeutic approaches.

**Method:**

We utilized the summary-data-based Mendelian randomization (SMR) method to investigate the causal relationships between druggable genes that encode plasma proteins and endometriosis. The data sources included the deCODE database, the UKB-PPP, and the FinnGen database. Colocalization analysis was used to identify whether candidate genes and the disease share a common causal genetic variant. Finally, we measured the protein abundance and relative mRNA expression levels of targeted druggable genes in the plasma and peripheral blood mononuclear cells (PBMCs) of endometriosis patients using ELISA and RT-qPCR.

**Results:**

By integrating the results of SMR and colocalization analyses, we found that *EPHB4* is strongly associated with the risk of endometriosis, with higher levels of *EPHB4* correlating with an increased risk of the condition (P_FDR_ < 0.05, PPH4 = 0.99). *RSPO3* is moderately associated, with higher levels of *RSPO3* correlating with an increased risk of endometriosis (P_FDR_ < 0.001, PPH4 = 0.78). *CD109*, *SAA1*, *SAA2*, *FSHB*, and *SEZ6L2* are weakly associated with endometriosis, with higher levels of *FSHB* and *SEZ6L2* correlating with an increased risk of endometriosis, and higher levels of *CD109*, *SAA1*, and *SAA2* correlating with a decreased risk of endometriosis (P_FDR_ < 0.05, PPH4 < 0.6). ELISA and RT-qPCR analyses showed that the EPHB4 protein abundance in plasma and mRNA expression levels in PBMCs were significantly higher in the endometriosis group compared to the control group (P-value < 0.05).

**Conclusions:**

We found that the druggable genes *EPHB4*, *CD109*, *SAA1*, *SAA2*, *FSHB*, and *SEZ6L2* may be associated with the pathogenesis of endometriosis and are potential therapeutic targets for drug treatment. However, this preliminary study is limited by sample size and population diversity, requiring further validation to confirm the reliability of these findings.

**Supplementary Information:**

The online version contains supplementary material available at 10.1186/s41065-025-00457-w.

Endometriosis is a prevalent disease affecting 5–10% of women of reproductive age worldwide [1]. It is a chronic, inflammatory gynecological condition, clinically defined by the presence of endometrial-like tissue outside the uterus. The condition often results in debilitating pain and other distressing symptoms [1, 2]. The majority of patients diagnosed with endometriosis typically require long-term pharmacological intervention or surgical treatment, with the objective of providing symptomatic relief [3]. Therefore, there is an urgent need to identify new pathogenic mechanisms and therapeutic targets.

*EPHB4* is a member of the Eph receptor family of transmembrane tyrosine kinases and plays an essential role in vascular development and angiogenesis [4, 5]. *EPHB4* plays a critical role in tumor proliferation, metastasis, and angiogenesis. Its overexpression has been associated with multiple malignancies, including prostate, breast, ovarian, uterine, and colorectal cancers, making it a promising target for anticancer drug development [4]. Moreover, studies have shown that the use of *EPHB4* inhibitors effectively suppresses angiogenesis and growth of endometriotic lesions, significantly reducing vascular density within the lesions, thereby delaying their progression [6].

Mendelian randomization(MR) uses genetic variations to establish causal relationships between risk factors and outcomes [7]. The growing accessibility of large-scale GWAS and molecular quantitative trait loci(QTL) data enables the investigation of the causal associations between genes and endometriosis at the expression and protein abundance levels [8].

In this study, we applied Mendelian randomization to explore the causal relationship between the druggable genome and endometriosis, with subsequent validation of target genes in clinical samples. Our goal was to identify promising therapeutic targets for drug intervention, investigate suitable pharmacological agents for targeted therapy, and provide a theoretical basis for advancing endometriosis treatment.

## Materials and methods

### Data sources of pQTL

**Fig. 1:**
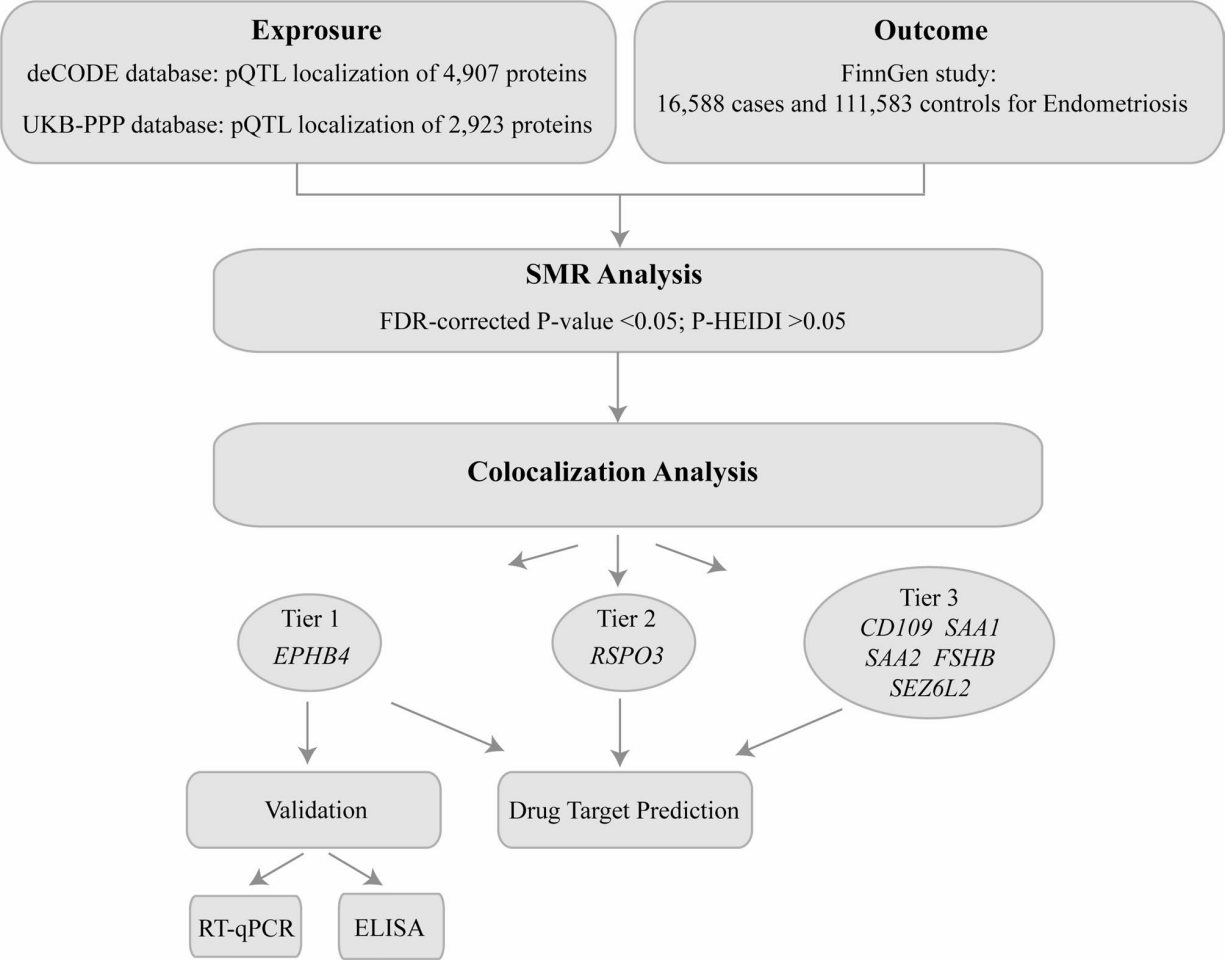
Flowchart of investigation

The key steps and detailed workflow of this study are presented in Fig. 1. We obtained summary statistics of the genetic associations with plasma protein levels from a study of Ferkingstad et al. (https://www.decode.com/summarydata/) [9] and the UK Biobank Pharma Proteomics Project (UKB-PPP)( https://registry.opendata.aws/ukbppp/) [10]. The study by Ferkingstad et al. included 35,559 Icelanders and adjusted for age, sex, and sample age for each tested protein, performing comprehensive pQTL localization of 4,907 proteins. The UK Biobank Pharma Proteomics Project is a collaboration between the UK Biobank (UKB) and thirteen biopharmaceutical companies. This project examined the plasma proteomic profiles of 54,219 UKB participants, performed comprehensive pQTL localization of 2,923 proteins, and identified a total of 14,287 significant genetic associations. (Table 1)

**Table 1:** Information of included studies and consortia

Exposure/Outcome	Consortium/First author	Participants
pQTL	deCODE GENETICS/Ferkingstad, E. et al.	35,559 Icelanders
	UKB-PPP	54,219 UKB participants
Endometriosis	The FinnGen study (Release 10)	16,588 European ancestry cases, 111,583 European ancestry controls

### The GWAS data of endometriosis

The outcome data used in this study were obtained from the FinnGen study (Release 10)( https://www.finngen.fi/en) [11], included 16,588 cases and 111,583 controls for endometriosis, which come from the European population.

### Druggable genes

The druggable genes involved in this study were derived from the study of Finan et al. [12], which identified a total of 4,479 druggable genes. Among them, 1,427 genes are targets of approved drugs or drugs in clinical development; 682 genes encode proteins closely related to known drug targets or drug-like compounds; and 2,370 genes represent extracellular proteins or members of key drug target families [12].

### Summary-data-based MR analysis

Summary-data-based Mendelian randomization (SMR) was employed to estimate the association of druggable genome protein abundance with the risk of endometriosis [13]. To select the most associated pQTL, a p-value threshold of 5.0E-8 was used for the SMR analysis [13]. The Heterogeneity in Dependent Instruments (HEIDI) method is a sensitivity analysis approach used to assess whether the Wald ratio estimates from multiple SNPs in a region exhibit greater heterogeneity than expected by chance, distinguishing effects mediated by linkage disequilibrium with a single causal variant from pleiotropy [13]. Associations with a P_HEIDI_ ≤ 0.05 in the HEIDI test were considered likely due to pleiotropy and were excluded from subsequent analyses. The Benjamini-Hochberg method was applied to adjust P-values from the SMR analysis, controlling the false discovery rate (FDR) at 0.05, to identify statistically significant and reliable genes, thereby enhancing the robustness of our conclusions. Associations with FDR-corrected P-value < 0.05 and P_HEIDI_ >0.05 were retained for further analysis [8].

### Colocalization analysis

Colocalization analysis was performed using the “coloc” package in R (version 4.3.1) to investigate shared causal variants between detected genes and endometriosis. Five different posterior probabilities corresponding to the colocalization analysis: H0: no causal variant for either trait; H1: Association with trait 1, not with trait 2; H2: Association with trait 2, not with trait 1; H3: Association with trait 1 and trait 2, two independent SNPs; H4: Association with trait 1 and trait 2, one shared SNP [14]. According to published articles, the colocalization region window was set at ± 1000 kb [15]. We set the prior probability that SNP is only associated with p1 or p2 at 1 × 10^− 4^. The posterior probability of hypothesis 4 (PPH4) was used to assess the degree of colocalization. The probability that the SNP is associated with both p1 and p2 is 1 × 10^− 5^. Based on previous studies [14, 16], we considered PPH4 > 0.8 as the threshold supporting high-level evidence of colocalization.

### Integrating results of SMR analysis and colocalization analysis

In order to gain a comprehensive understanding of the association between druggable genome and endometriosis at different levels, we integrated the results of previous analyses. The causal candidate genes were divided into different tiers according to the following criteria: (1) Tier 1 genes were defined as having gene-endometriosis associations at the protein abundance level in both the deCODE study and the UKB-PPP study (P-value < 0.05), and with high-level evidence of colocalization (PPH4 > 0.8). (2) Tier 2 genes were defined as having gene-endometriosis associations at the protein abundance level in the deCODE study or the UKB-PPP study (P-value < 0.05), and with moderate-level evidence of colocalization (0.6 < PPH4 ≤ 0.8). (3) Tier 3 genes were defined as having gene-endometriosis associations at the protein abundance level in the deCODE study or the UKB-PPP study (P-value < 0.05), and with low-level evidence of colocalization (PPH4 ≤ 0.6).

### Drug target prediction

The DRUGBANK database contains FDA-approved drugs, drugs currently in clinical trials, and experimental drugs, supplying detailed information on chemical structures, pharmacological properties, mechanisms of action, and drug interactions [17]. Drug prediction was performed on the druggable genes screened in previous steps using the DRUGBANK database.

### Clinical sample collection

We recruited 24 study participants, with the case group consisting of 12 patients diagnosed with endometriosis at the outpatient clinic of the Affiliated Hospital of Youjiang Medical University for Nationalities, and the control group comprising 12 non-endometriosis patients. All participants were free from hormonal therapy or contraceptive use for at least three month prior to blood sampling. Patients in the endometriosis group underwent laparoscopic examination prior to recruitment, and the postoperative pathology confirmed the diagnosis of endometriosis. The control group exhibited no clinical symptoms related to endometriosis, and ultrasound examinations revealed no abnormal lesions. Our study was approved by the Youjiang Medical University for Nationalities Ethics Committee and was agreed to by all participants with signed informed consent.

Fasting peripheral venous blood was collected from participants in the control and endometriosis groups into two separate tubes: one containing sodium citrate as an anticoagulant for plasma extraction, and the other containing EDTA for peripheral blood mononuclear cells (PBMCs) isolation. After centrifugation at 3000 rpm for 10 min, plasma was obtained from the tube containing sodium citrate as an anticoagulant. EDTA-anticoagulated blood was diluted 1:1 with PBS and then added to lymphocyte separation medium. After centrifugation, the intermediate buffy coat layer was collected into an EP tube and washed twice with PBS to isolate PBMCs.

### ELISA analysis

Sandwich ELISA kits from Byabscience Biotechnology (Catalogue number: BY-EH112633) were employed for detecting EPHB4. All assays were performed in strict accordance with the manufacturer’s protocols. Absorbance was measured at 450 nm using the Rayto RT-6100 Enzyme Labeling System, and protein concentration was determined by calculating the average absorbance of each sample based on the standard curve dedicated to each measured protein.

### RT-qPCR analysis

We isolated total RNA from PBMCs using TRIzol reagent. The total RNA was reverse transcribed into cDNA using ToloScript All-in-one RT EasyMix for qPCR (Tolobio, China, Catalogue number: #22107), and all steps followed the manufacturer’s protocol. Finally, qPCR was conducted using 2 × Q3 SYBR qPCR Master mix (Universal) (Tolobio, China, Catalogue number: #22204). The primers were as follows: *EPHB4*: forward(5′-GAACATCACAGCCAGACCCAAC-3′); and reverse (5′-CACCAGGACCAGGACCACAC-3′). *GAPDH* was used as the internal reference gene for the total RNA. The relative expression of targeted genes was calculated using the 2^−△△CT^ method.

### Statistical analysis

Statistical analysis and result visualization in this study were performed using R (4.4.0) and GraphPad Prism (9.4.1). Quantitative data following a normal distribution were analyzed using the t-test and presented as mean ± standard deviation. For non-normally distributed quantitative data, the Mann-Whitney U test was applied, with results expressed as median (minimum, maximum). P-value < 0.05 was considered statistically significant.

## Results

### Results of SMR and colocalization analyses

**Fig. 2:**
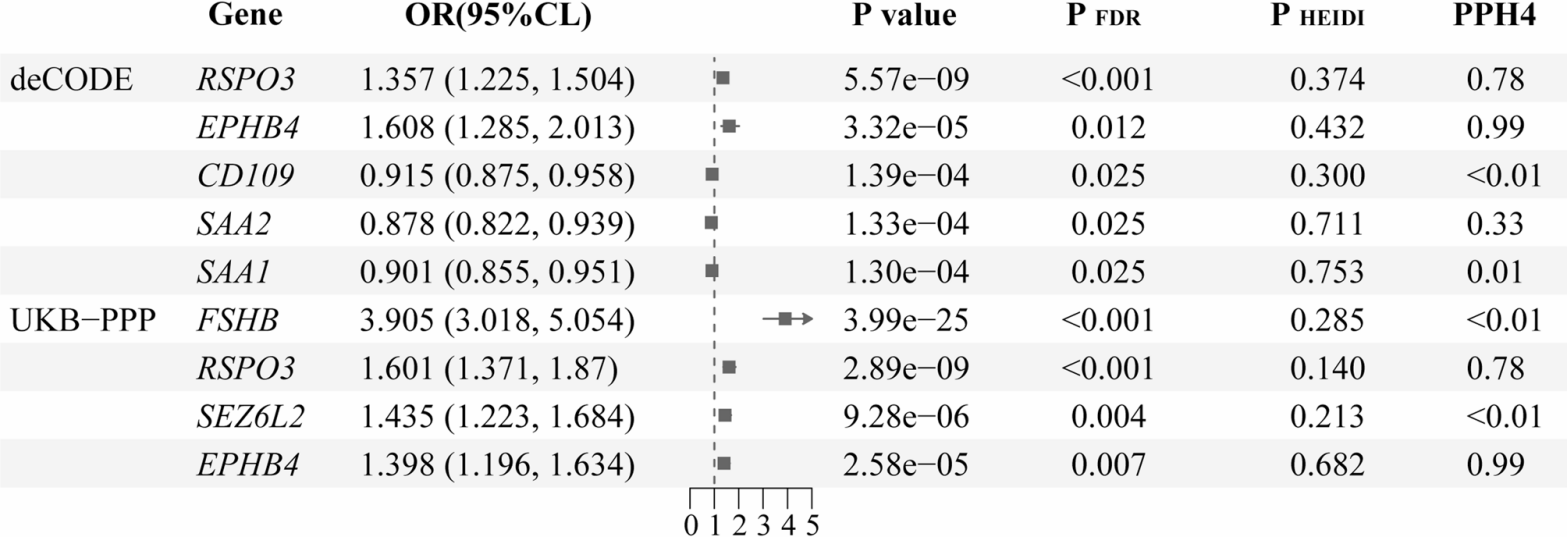
Associations of genetically predicted gene encoded protein with endometriosis in Mendelian randomization analysis

Using SMR analysis, we assessed the association between 1,002 and 1,146 druggable genomes and endometriosis in the deCODE and UKB-PPP studies, respectively (Table S1–S2). Subsequently, we adjusted the P-values using the Benjamini-Hochberg method and screened for genes meeting the criteria of adjusted P-value < 0.05 and PHEIDI > 0.05. As a result, we identified that RSPO3(OR 1.357, 95%CI 1.225–1.504), EPHB4(OR 1.608, 95%CI 1.285–2.013), SAA1(OR 0.901, 95%CI 0.855–0.951), SAA2(OR 0.878, 95%CI 0.822–0.939), and CD109(OR 0.915, 95%CI 0.875 − 0.958) were associated with endometriosis at the protein abundance level in the deCODE study, while FSHB(OR 3.905, 95%CI 3.018–5.054), RSPO3(OR 1.601, 95%CI 1.371–1.87), SEZ6L2(OR 1.435, 95%CI 1.223–1.684), and EPHB4(OR 1.398, 95%CI 1.196–1.634) were associated with endometriosis at the protein abundance level in the UKB-PPP study. (Fig. 2)

After colocalization analysis, we identified that *EPHB4* is considered to support high-level evidence of colocalization with endometriosis (PPH4 = 0.993), while *RSPO3* is considered to support moderate-level evidence of colocalization with endometriosis (PPH4 = 0.783). *CD109*, *SAA1*, *SAA2*, *FSHB*, and *SEZ6L2* are considered to provide low-level evidence of colocalization with endometriosis (PPH4 ≤ 0.6). (Table 2)

**Table 2:** Tier of genetically predicted protein of candidate gene with endometriosis in MR

Gene	Tier	deCODE			UKB-PPP			Colocalization Analysis
		OR (95%CI)	*P*-FDR		OR (95%CI)	*P*-FDR		PPH4
*EPHB4*	Tier 1	1.608 (1.285, 2.013)	0.012		1.398 (1.196, 1.634)	0.007		0.99
*RSPO3*	Tier 2	1.357 (1.225, 1.504)	< 0.001		1.601 (1.371, 1.87)	< 0.001		0.78
*CD109*	Tier 3	0.915 (0.875, 0.958)	0.025		0.97 (0.941, 0.999)	0.529		0.33
*SAA1*	Tier 3	0.901 (0.855, 0.951)	0.025		\	\		< 0.01
*SAA2*	Tier 3	0.878 (0.822, 0.939)	0.025		\	\		0.01
*FSHB*	Tier 3	\	\		3.905 (3.018, 5.054)	< 0.001		< 0.01
*SEZ6L2*	Tier 3	\	\		1.435 (1.223, 1.684)	0.004		< 0.01

### Integration of SMR and colocalization analyses results

By integrating the results of SMR and colocalization analysis, *EPHB4* was identified as a tier 1 gene, *RSPO3* as a tier 2 gene, and *CD109*, *SAA1*, *SAA2*, *FSHB*, and *SEZ6L2* as tier 3 genes (Table 2). Meanwhile, our study suggested that higher levels of *EPHB4*, *RSPO3*, *FSHB*, and *SEZ6L2* were associated with an increased risk of endometriosis, while lower levels of *CD109*, *SAA1*, and *SAA2* were also associated with an increased risk of endometriosis (Table 2).

The integration of MR and colozalization data allows us to conclude that *EPHB4* is a Tier 1 gene, with the strongest evidence supporting its association with the pathogenesis of endometriosis. Therefore, we validated the expression of *EPHB4* at both protein abundance and mRNA levels in peripheral blood.

### EPHB4 protein and gene expression levels in plasma and PBMCs

The clinical information of the patients recruited for the study can be found in Table 3. We observed that a comparison between the control group and the endometriosis group revealed a statistically significant difference in BMI levels (*P* < 0.05). This aligns with the findings of several previous studies [18, 19].

**Table 3:** The clinical and biochemical characteristics of controls and Endometriosis patients who participated in this study

	Control (*n* = 12)	Endometriosis (*n* = 12)	*P-value*	
Age (years)	33.4 ± 4.66	34.3 ± 3.41	0.623	
BMI (kg/m2)	24.5 ± 3.91	20.8 ± 2.68	0.0137	
AMH (ng/mL)	3.38 ± 3.19	2.78 ± 1.42	0.561	
FSH (IU/L)	8.19 ± 3.94	5.24 ± 3.44	0.0635	
LH (IU/L)	5.30 (1.68, 19.1)	2.74 (0.240, 10.6)	0.0916	
LH/FSH ratio	1.38 (0.378, 7.25)	1.46 (0.658, 10.3)	0.592	
PRL (ug/L)	0.321 ± 0.127	0.339 ± 0.187	0.782	
E2 (pg/mL)	35.1 (5.00, 214)	24.6 (5.00, 61.2)	0.138	
PROG (ng/mL)	0.321 ± 0.127	0.339 ± 0.187	0.782	
T (ug/L)	0.223 ± 0.110	0.265 ± 0.0955	0.324	
Menarche Age (years)	13.5 (12.0, 16.0)	13.0 (11.0, 15.0)	0.709	

Data are presented as the median (minimum, maximum) or as the mean ± standard deviation.

AMH Anti Mullerian Hormone, BMI Body Mass Index; FSH Follicle Stimulating Hormone; LH Luteinizing Hormone; E2 Estradiol; PRL Prolactin; PROG Progesterone; T Testosterone.

**Fig. 3:**
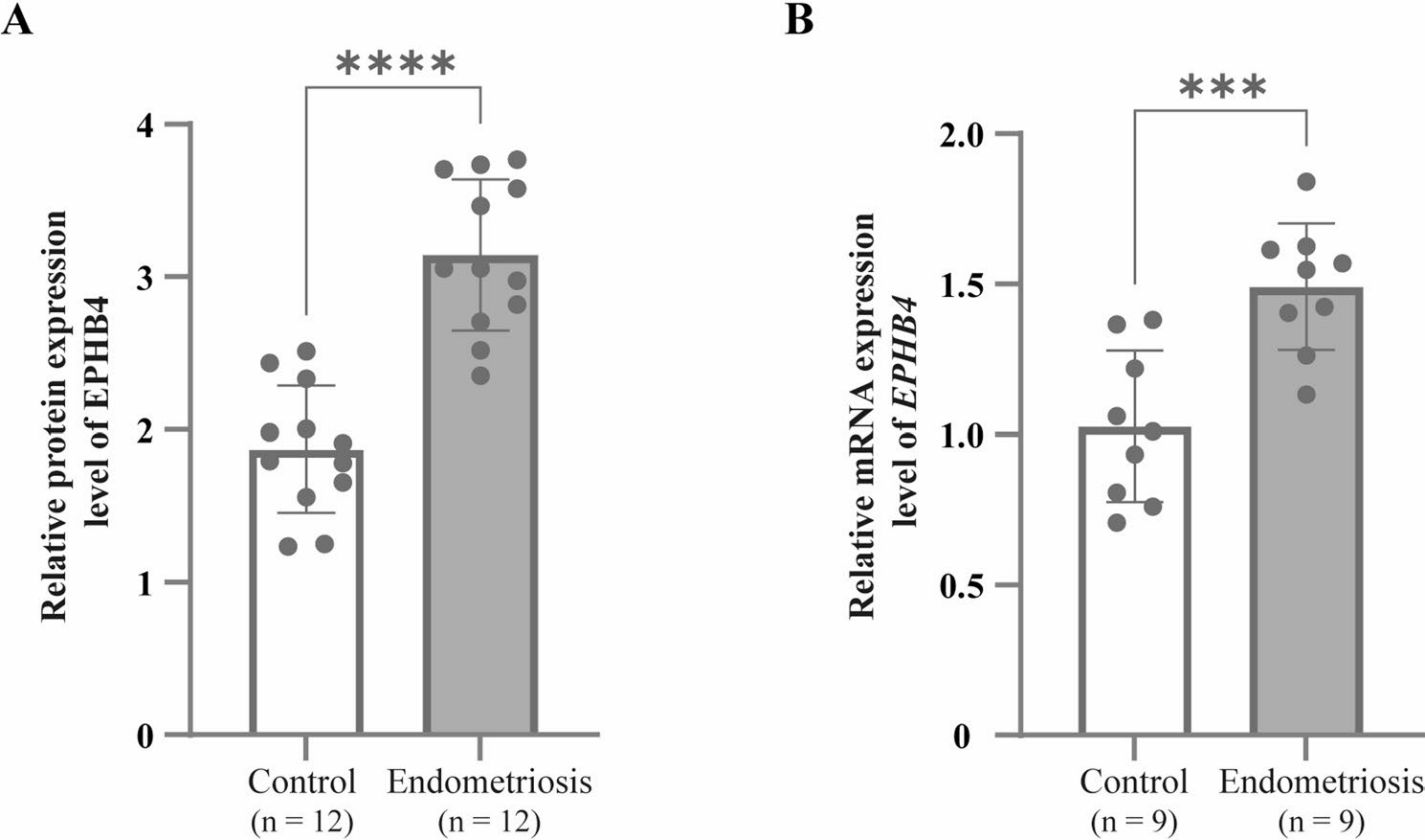
Expression levels of EPHB4 in control and endometriosis groups. (**A**) Relative protein expression levels of EPHB4 in plasma. (**B**) Relative mRNA expression levels of EPHB4 in PBMCs. ***P < 0.001, ****P < 0.0001

The protein expression level of EPHB4 in plasma from 12 patients with endometriosis and 12 controls was assessed using ELISA. The results showed that the relative protein expression level of EPHB4 in plasma was significantly higher in the endometriosis group compared to the control group (*P* < 0.05). During mRNA extraction from PBMCs of 24 patients, some samples were excluded due to insufficient total RNA concentration or inadequate purity. Consequently, usable mRNA was successfully obtained from only 18 samples. RT-qPCR analysis showed that the relative mRNA expression level of *EPHB4* in PBMCs was significantly higher in the endometriosis group compared to the control group (*P* < 0.05). (Fig. 3)

### Drug target prediction

We investigated the potential drugs related to the target proteins in the DRUGBANK database by utilizing the seven druggable targets obtained in the previous step. Proteins encoded by *EPHB4* were targeted by 11 drugs (DB01254, DB07251, DB07253, DB07256, DB07254, DB11973, DB07249, DB07250, DB07252, DB07255, DB12010), suggesting that these pharmacological interventions may facilitate the treatment of endometriosis through the modulation of *EPHB4*. *RSPO3*, *CD109*, *SAA1*, *SAA2*, *FSHB*, and *SEZ6L2* had no available corresponding drugs, indicating that these proteins are potential targets for drug discovery. (Table S3)

## Discussion

In this study, we analysed the causal relationship between plasma proteins and endometriosis using publicly available large-sample pQTL and GWAS databases, and identified 7 proteins associated with the risk of endometriosis. By integrating the results of SMR and colocalization analyses, we found that *EPHB4* had a high level of correlation with the risk of developing endometriosis, while *RSPO3* showed a moderate level of correlation. In contrast, *CD109*, *SAA1*, *SAA2*, *FSHB*, and *SEZ6L2* exhibited low levels of correlation with this risk.

Angiogenesis is essential for normal tissue growth, development, and repair. An imbalance between pro-angiogenic and anti-angiogenic signals during this process can lead to various pathological conditions [20]. Receptor tyrosine kinases (RTKs), including vascular endothelial growth factor receptors (VEGFRs), angiopoietin receptors, and *EPHB4* along with its ligand EphrinB2, have been identified as key mediators of angiogenesis [20]. *EPHB4* and its ligand EphrinB2 play a critical role in angiogenesis during embryonic vascular development—including the formation, stabilization, branching, remodeling, and arteriovenous differentiation of immature vessels—as well as in physiological and pathological processes such as wound healing, tumorigenesis, and ocular neovascularization [20]. Studies have shown that, angiogenesis is a crucial step in the pathogenesis of endometriosis, as endometriotic lesions require the formation of new blood vessels [21, 22], The survival of endometriotic lesions is contingent upon the presence of an adequate blood supply [6, 23]. A study conducted by Gülen Yerlikaya et al. involving 61 patients with endometriosis and 53 controls found that the mRNA expression levels of *EPHB4* are significantly higher in ectopic lesions compared to normal tissues, with the highest levels observed in peritoneal implants [24]. The study by Rudzitis-Auth J et al. confirmed that the *EPHB4* inhibitor NVP-BHG712 suppressed the migration, tube formation, and sprouting activity of both human dermal microvascular endothelial cells and mouse aortic rings, thereby inhibiting the vascularization and growth of endometriotic lesions [6]. Research suggests that angiogenesis in endometriotic lesions may be mediated by angiogenic factors such as stromal cell-derived factor (SDF-1) and its receptor, chemokine receptor type 4 (CXCR4) [25]. *EPHB4* activation promotes SDF-1-induced chemotaxis in endothelial cells [26]. By using the *EPHB4* inhibitor NVP-BHG712, the expression of *SDF-1* and *CXCR4* in the area vasculosa of chicken embryos is downregulated [27]. Thus, *EPHB4* may mediate the pathogenesis of endometriosis through *SDF-1/CXCR4*-driven angiogenesis [6]. This is consistent with our research, which found that *EPHB4* was strongly correlated with the risk of endometriosis using Mendelian randomization analysis. To further substantiate our hypothesis, we conducted tests at both the transcriptional and protein abundance levels. The results showed that both the mRNA expression levels of EPHB4 in PBMCs and the protein abundance of EPHB4 in plasma were significantly elevated in patients with endometriosis compared to the control group. Therefore, *EPHB4* represents a promising therapeutic target for the treatment of endometriosis. However, further studies are needed to validate this hypothesis.

In addition, we used the DRUGBANK database to obtain a list of drugs that target EPHB4. Among these drugs, Dasatinib [28] and Tesevatinib [29] can act on EPHB4, involved in regulating cell adhesion and migration, and play important roles in cardiac morphogenesis, angiogenesis, vascular remodelling and permeability. Dasatinib has been approved by the FDA for the treatment of Philadelphia chromosome-positive (Ph+) chronic myelogenous leukemia and Ph + acute lymphoblastic leukemia [30]. A phase II clinical trial evaluated the efficacy of Dasatinib as a monotherapy for recurrent or persistent ovarian cancer, fallopian tube cancer, primary peritoneal cancer, and endometrial cancer. The results showed that 71% (*n* = 20) of patients experienced grade 3 adverse events, with anemia (*n* = 8, 28.6%), fatigue (*n* = 7, 25%), and dyspnea (*n* = 5, 17.9%) being the most common. Additionally, 7.1% of patients developed grade 4 anemia [30]. Tesevatinib has been evaluated in clinical trials for its potential to treat non-small cell lung cancer, with the most common treatment-related adverse events being grade 1 and 2 diarrhea, rash, nausea, and fatigue [31, 32]. However, there are currently no studies on the use of these drugs in the treatment of endometriosis.

Endometriosis is a disease characterized by the growth of ectopic endometrial tissue. Angiogenesis plays an indispensable role in the formation and progression of endometriotic lesions, with endothelial cells (EC) being critical in neovascularization [33]. *R-spondin 3* (*RSPO3*) is a secreted protein primarily produced by endothelial cells, with studies demonstrating its protective effects on these cells [34]. After inflammatory vascular injury, *RSPO3* derived from endothelial cells promotes endothelial regeneration by activating the β-catenin and *ILK* signaling pathways in an *LGR4*-dependent manner [34]. Therefore, we speculate that *RSPO3* may affect endothelial cells and thus play a role in the pathogenesis of intrauterine endometriosis. In addition, studies have found that estradiol can upregulate RSPO3 [35], suggesting a potential association between *RSPO3* and endometriosis, as endometriosis is an estrogen-dependent disease in which estrogen plays a crucial role throughout its progression [36]. However, clinical and basic research on the relationship between *RSPO3* and endometriosis remains lacking, necessitating further studies to explore their association.

*CD109* is a glycosylphosphatidylinositol (GPI)-anchored protein that regulates the TGF-β and NF-κB signaling pathways, thereby influencing immune responses and inflammation [37]. *CD109* may play a role in maintaining immune homeostasis and alleviating inflammatory diseases by regulating the function of specific immune cells [37]. Studies have found that in transgenic mice with overexpression of *CD109* in the epidermis, macrophage and neutrophil recruitment, as well as the granulation tissue area, are significantly reduced in the wound [38]. Therefore, *CD109* may help prevent excessive inflammation and autoimmunity, playing a key role in maintaining immune balance and reducing the risk of inflammatory diseases [37].The inflammatory nature of endometriosis has been well established over the years [39]. In this study, we found that low expression of *CD109* may be associated with an increased risk of endometriosis. Therefore, we speculate that *CD109* may be involved in the pathogenesis of endometriosis by regulating the inflammatory response.

*SAA1* and *SAA2* are members of the SAA family and are the most prominent members of the acute phase response (APR), which is closely associated with the inflammatory response [40, 41]. The results of our study indicate that a low expression of *SAA1* and *SAA2* is associated with an increased risk of endometriosis. This may be related to inflammatory response; however, there are currently no reports of this. FSHB is responsible for encoding the beta subunit of follicle-stimulating hormone [42]. It has been shown that polymorphisms in the *FSHB* gene play a role in the pathogenesis of endometriosis [43, 44]. This aligns with our findings. The results are consistent with those of our previous research.

In a previous study, Tian Tao et al. used MR analysis on plasma proteins from the UKB-PPP to investigate druggable genome linked to endometriosis, identifying *FSHB*, *RSPO3*, *SEZ6L2*, and *EPHB4* as potential therapeutic targets [45]. Our study expanded the research dataset by incorporating plasma protein data from the deCODE database. Subsequent analysis revealed *EPHB4* as the druggable genome with the highest level of supporting evidence. Finally, we validated the *EPHB4* mRNA expression level and protein abundance in peripheral blood from clinical samples, further confirming the association between *EPHB4* and the pathogenesis of endometriosis.

Our study has several strengths. Due to the random allocation of genetic variants at conception, potential confounding factors that may influence endometriosis—such as endometrial implantation, inflammatory responses, and surgical procedures—cannot alter these genetic variants. Therefore, compared with traditional observational studies, the MR approach is less susceptible to confounding bias and reverse causation. By integrating pQTL data from multiple databases to enhance the credibility of the results, and further validating our findings through experimental verification using clinical samples, we provided strong support for the conclusions of this study.

This study has several limitations. Firstly, the database used primarily focuses on populations of European descent, which may limit the applicability of the findings to other populations and ethnic groups. Secondly, although colocalization analysis ruled out bias caused by linkage disequilibrium, horizontal pleiotropy could not be minimized. The HEIDI test with a conservative P-value threshold may help mitigate its impact. Additionally, this study focused on the analysis and validation of plasma proteins; however, further validation in endometriotic lesion tissues was not performed, which may to some extent affect the applicability of the findings at the tissue level. Lastly, while potential drugs for treating endometriosis, such as EPHB4 inhibitors, were discussed, experimental validation in endometriosis models has not been conducted.

## Conclusions

This study indicates that the druggable genes *EPHB4*, *CD109*, *SAA1*, *SAA2*, *FSHB*, and *SEZ6L2* may be associated with the pathogenesis of endometriosis and are potential therapeutic targets for drug treatment. Validation using clinical samples further confirmed that *EPHB4* mRNA expression in PBMCs and protein abundance in plasma were significantly elevated in patients with endometriosis compared to controls. This study presents potential therapeutic targets for pharmacological intervention in endometriosis, though additional research is essential to confirm their efficacy.

## Electronic supplementary material

Below is the link to the electronic supplementary material.

Supplementary Material 1

## Data Availability

No datasets were generated or analysed during the current study.

## Abbreviations

ELISA: Enzyme-Linked Immunosorbent Assay
FDR: False Discovery Rate
HEIDI: Heterogeneity in the Dependent Instrument
MR: Mendelian randomization
PBMCs: Peripheral Blood Mononuclear Cells
PPH4: Posterior probability of H4
pQTL: Protein quantitative trait loci
RT-qPCR: Reverse Transcription Quantitative PCR
SD: Standard Deviation
SMR: Summary-data-based Mendelian Randomization
SNP: Single Nucleotide Polymorphism
UKB: UK Biobank
UKB-PPP: UK Biobank Pharma Proteomics Project
